# Glucose-Dependent Insulinotropic Polypeptide Suppresses Foam Cell Formation of Macrophages through Inhibition of the Cyclin-Dependent Kinase 5-CD36 Pathway

**DOI:** 10.3390/biomedicines9070832

**Published:** 2021-07-16

**Authors:** Michishige Terasaki, Hironori Yashima, Yusaku Mori, Tomomi Saito, Yoshie Shiraga, Raichi Kawakami, Makoto Ohara, Tomoyasu Fukui, Tsutomu Hirano, Yuichiro Yamada, Yutaka Seino, Sho-ichi Yamagishi

**Affiliations:** 1 Department of Medicine, Division of Diabetes, Metabolism, and Endocrinology, Showa University School of Medicine, Tokyo 142-8666, Japan; yashima@med.showa-u.ac.jp (H.Y.); saito_to@cnt.showa-u.ac.jp (T.S.); yoshie.shiraga@gmail.com (Y.S.); 11112030m@gmail.com (R.K.); s6018@nms.ac.jp (M.O.); showauft@med.showa-u.ac.jp (T.F.); shoichi@med.showa-u.ac.jp (S.-i.Y.); 2 Anti-Glycation Research Section, Department of Medicine, Division of Diabetes, Metabolism, and Endocrinology, Showa University School of Medicine, Tokyo 142-8666, Japan; u-mori@med.showa-u.ac.jp; 3 Diabetes Center, Ebina General Hospital, Ebina 243-0433, Japan; hirano@med.showa-u.ac.jp; 4 Kansai Electric Power Medical Research Institute, Osaka 553-0003, Japan; yamada.yuuichiro@a3.kepco.co.jp (Y.Y.); yutaka.seino.hp@gmail.com (Y.S.)

**Keywords:** GIP, CD36, Cdk5, GIP receptor, macrophages

## Abstract

Glucose-dependent insulinotropic polypeptide (GIP) has been reported to have an atheroprotective property in animal models. However, the effect of GIP on macrophage foam cell formation, a crucial step of atherosclerosis, remains largely unknown. We investigated the effects of GIP on foam cell formation of, and *CD36* expression in, macrophages extracted from GIP receptor-deficient (*Gipr^−/−^*) and *Gipr^+/+^* mice and cultured human U937 macrophages by using an agonist for GIP receptor, [D-Ala^2^]GIP(1–42). Foam cell formation evaluated by esterification of free cholesterol to cholesteryl ester and *CD36* gene expression in macrophages isolated from *Gipr^+/+^* mice infused subcutaneously with [D-Ala^2^]GIP(1–42) were significantly suppressed compared with vehicle-treated mice, while these beneficial effects were not observed in macrophages isolated from *Gipr^−/−^* mice infused with [D-Ala^2^]GIP(1–42). When macrophages were isolated from *Gipr^+/+^* and *Gipr^−/−^* mice, and then exposed to [D-Ala^2^]GIP(1–42), similar results were obtained. [D-Ala^2^]GIP(1–42) attenuated ox-LDL uptake of, and *CD36* gene expression in, human U937 macrophages as well. Gene expression level of cyclin-dependent kinase 5 (*Cdk5*) was also suppressed by [D-Ala^2^]GIP(1–42) in U937 cells, which was corelated with that of *CD36*. A selective inhibitor of Cdk5, (*R*)-DRF053 mimicked the effects of [D-Ala^2^]GIP(1–42) in U937 cells. The present study suggests that GIP could inhibit foam cell formation of macrophages by suppressing the Cdk5-CD36 pathway via GIP receptor.

## 1. Introduction

Cardiovascular disease (CVD) is one of the devasting complications in diabetes and accounts for the increased risk of mortality in these patients all over the world [1,2]. Indeed, the Emerging Risk Factors Collaboration study revealed that after adjustment for traditional coronary risk factors, the risk of death from cardiovascular causes increased by 2.3-fold in patients with diabetes compared with non-diabetic subjects [1].

In the subendothelial space, low-density lipoprotein (LDL) is changed to oxidized LDL (ox-LDL) by oxidative modifications of apolipoprotein B100, which could stimulate adhesion molecule and chemokine expression within the atherosclerotic plaques, thereby promoting esterification of free cholesterol to cholesteryl ester and foam cell formation of macrophages, one of the initial characteristic features of atherosclerotic CVD [3,4,5]. Scavenger receptor CD36 has been shown to contribute to ox-LDL uptake by macrophages and subsequent foam cell formation within the atherosclerotic lesions [3,5].

Glucose-dependent insulinotropic polypeptide (GIP), which is one of the incretins produced by K-cells in the small intestine in response to lipids and/or sugars, has been known to promote secretion of insulin in a glucose-dependent manner [6,7,8,9]. Besides its blood glucose-lowering action, GIP has been reported to have atheroprotective actions in animal models [10,11,12,13,14,15]. Indeed, we have previously reported that chronic infusion of active GIP(1–42) attenuates aortic plaque formation in apolipoprotein E-null (*Apoe^−/−^*) mice, whose actions were totally independent of blood pressure, body weight, food intake, and plasma lipid or glucose levels [14,15]. Furthermore, ex vivo-treatment with active GIP(1–42) has also been found to suppress the foam cell formation of, and *CD36* gene expression in, macrophages isolated from *Apoe^−/−^* mice [14,15]. However, the underlying molecular mechanism for the inhibitory effects of GIP on foam cell formation of macrophages remains largely unknown. In other words, how GIP could inhibit foam cell formation of, and *CD36* gene expression in, macrophages are not sufficiently understood.

Cyclin-dependent kinases (Cdks) have principal roles in regulation of cell cycle, transcription, and differentiation [16,17]. Cyclin-dependent kinase 5 (Cdk5) is considered to be unique because in contrast to other Cdk members, Cdk5 is not a modulator of cell cycle procession [18,19,20], but a regulator of gene modulation and cell survive [21]. Cdk5 could phosphorylate lysine–serine–proline motif of neurofilaments, which plays a crucial role in neuronal cell development, differentiation and migration in supernumerary spinal and cranial motor neurons, while its functional disorder is involved in neurodegenerative disorders, such as Alzheimer’s disease [21,22,23]. Recently, Cdk5 has been reported to contribute to endothelial cell senescence [24], and truncated regulatory subunit of Cdk5 has been shown to be accumulated within the atherosclerotic lesions, and long-term suppression of Cdk5 attenuates the progression of atherosclerosis in *Apoe^−/−^* mice by reducing the inflammatory reactions [18]. Moreover, Cdk5 is abundantly expressed in macrophages and could mediate the lipopolysaccharide-induced inflammatory reactions [19]. Furthermore, we have recently found that advanced glycation end products (AGEs), aging molecules formed at an accelerated rate under diabetes, stimulate macrophage foam cell formation via the activation of Cdk5-CD36 pathway [25]. Since AGEs are localized in monocyte/macrophage-derived foam cells within the atherosclerotic plaques, macrophage foam cell formation evoked by AGEs could cause the atherosclerotic plaque instability and resultantly increase the risk of CVD in diabetes [26,27,28,29,30]. These observations suggest that the Cdk5-CD36 pathway in macrophages could be a therapeutic target for CVD. However as far as we know, there is no paper to show the effects of GIP on foam cell formation and Cdk5-CD36 pathway in macrophages. Therefore, we investigated here whether [D-Ala^2^]GIP(1–42), an agonist for GIP receptor, could inhibit the macrophage foam cell formation by suppressing the Cdk5-CD36 pathway via GIP receptor interaction by using macrophages extracted from [D-Ala^2^]GIP(1–42)-administrated GIP receptor-deficient (*Gipr^−/−^*) and *Gipr^+/+^* mice, [D-Ala^2^]GIP(1–42)-treated macrophages isolated from *Gipr^−/−^* and *Gipr^+/+^* mice, and human U937 macrophages.

## 2. Materials and Methods

### 2.1. Materials and Reagents

Materials and chemical regents were purchased as follows: [D-Ala^2^]GIP(1–42) from Phoenix Pharmaceuticals. Inc. (Burlingame, CA, USA), a human monocytic lymphoma line, U 937 cells from JCRB (JCRB9021; Osaka, Japan), phorbol 12-myristate 13-acetate (PMA) and Roswell Park Memorial Institute (RPMI) 1640 medium from Sigma Aldrich (St. Louis, MO, USA), 1,1′-dioctadecyl-3,3,3′,3′-tetamethylindocarbocyanine perchlorate (Dil)-ox-LDL from Highland Technology Center (Frederick, MD, USA), and a selective Cdk5 inhibitor, (*R*)-DRF053 was from R&D Systems, Inc. (Minneapolis, MN, USA).

### 2.2. Animal Experiments

The protocol and design of this experiments were approved by the Animal Care Committee of Showa University (permission number: 04141) and Akita University Graduate School of Medicine (approval number: a-1-2520). All experiments, surgeries or sacrifices, were conducted with efforts to minimize the suffering using general anesthesia of isoflurane. *Gipr^−/−^* and *Gipr^+/+^* mice were bred (backcrossed to C57BL/6J strain for >18 generations to minimize variability of gene) as described previously [31]. A total of 10 male *Gipr^−/−^* and *Gipr^+/+^* mice, respectively, at 7 weeks old were transferred from Akita University Graduate School of Medicine to Animal Institute of Showa University School. The mice were kept on a standard food with free water in the room controlled at 21 °C temperature, under a 12-h light and dark cycle and 40–60% humidity. At 9 weeks old, the mice were subcutaneously infused with [D-Ala^2^]GIP(1–42) at 25 nmol/kg/day or saline by osmotic mini-pumps. At 13 weeks old, we collected blood samples and peritoneal macrophages from the mice after intraperitoneal injection of thioglycolate broth as described previously [14,15,32,33,34,35,36]. Also, macrophages were first isolated from *Gipr^−/−^* and *Gipr^+/+^* mice at 21 weeks old, respectively, and then exposed to [D-Ala^2^]GIP(1–42) at 1 nmol/L for 18 h as described previously [14,15].

### 2.3. Characteristics and Biochemical Parameters in Mice

Blood samples collected after a 12-h fast were used for the evaluation of biochemical parameters. Food intake, body weight, heart rate, and systolic and diastolic blood pressure (SBP and DBP) were calculated, and total-cholesterol (Total-C), high-density lipoprotein cholesterol (HDL-C), triglycerides, insulin, Total-GIP, fasting blood glucose (FBG), glycated hemoglobin (HbA1c) levels were measured as described previously [14,15,32,33,34,35,36].

### 2.4. Cholesterol Esterification Assay of Macrophages Extracted from Mice

Cholesterol esterification assay was performed as described previously [14,15,32,33,34,35,36]. Peritoneal macrophages extracted from *Gipr^−/−^* and *Gipr^+/+^* mice were incubated with 10 μg/mL ox-LDL and 0.1 mmol/L [^3^H]oleate. After 18 h, cellular lipids were extracted and the radioactivity of cholesterol [^3^H] oleate was measured by a thin-layer chromatography.

### 2.5. Experiments of U937 Macrophages

U937 cells were cultured in RPMI 1640 medium containing 10% fetal bovine serum (FCS), 100 μg/mL streptomycin and 100 U/mL penicillin. The floating cells were seeded onto 24-well dishes and incubated with 40 ng/mL PMA in RPMI 1640 medium containing 10% FCS at 37 °C in a humidified atmosphere with 5% CO_2_ for 24 h. After twice rinsing gently by phosphate-buffer saline (PBS), adherent cells were prepared as differentiated macrophages. Previously, the adherent cells used like this experiment were differentiated from monocytes to macrophages by analysis of fluorescence-activated cell sorting (FACS) [25,32,36,37,38,39,40]. U937 macrophages were treated with or without 1 nmol/L [D-Ala^2^]GIP, or 0.215 µmol/L (*R*)-DRF053 in RPMI 1640 medium including 10% FCS at 37 °C in 5% CO_2_ for 18 h.

### 2.6. Dil-ox-LDL Uptake into Macrophages

The U937 cells were treated with 10 μg/mL Dil-ox-LDL in RPMI 1640 medium including 10% FCS at 37 °C in 5% CO_2_ for 18 h [25,32,36]. After twice washing with PBS gently, immunofluorescence was observed using Keyence BZ-X710 microscope and analyzed with the Keyence BZ-X710 software (Osaka, Japan). The quantification of fluorescent intensity of red color per cells was calculated as described previously [25,32,36].

### 2.7. Levels of Gene Expression

Levels of gene expression were determined by real-time RT-PCR using TaqMan or SYBR gene expression assay as described previously [14,15,32,33,34,35,36]. In brief, total RNA was extracted from the adherent macrophages to synthesize cDNA. Gene expression levels were initialized with glyceraldehyde 3-phosphate dehydrogenase (*GAPDH)* mRNA-derived intensities, and the data were expressed as relative levels to the controls. Probes and primers for mice were as follows: mouse; *Gipr*, Mm01316344_ml; *CD36*, Mm01135198_ml; *Gapdh*, Mm03302249_g1. Probes and primers for human were as follows; *CD36*, Hs00169627_ml; *Cdk5,* NM_001164410.3, NM_004935.4; *Gapdh*, Hs99999905_ml.

### 2.8. Statistical Analysis

Data were presented as mean ± SD. The statistical analyses above two groups were performed by appropriate ANOVA; Unpaired *t*-test was used to compare two groups. The correlation between two groups was analysed by Peason’s correlation test. All analyses were performed using PRISM (version 7.05, GraphPad Inc., San Diego, CA, USA). Differences were defined statistically significant at *p* < 0.05.

## 3. Results

### 3.1. Characteristics and Biochemical Data of Gipr^−/−^ Mice and Gipr^+/+^ Mice

Laboratory data of *Gipr^−/−^* and *Gipr^+/+^* mice infused with or without [D-Ala^2^]GIP(1–42) are presented in Table 1. There were no significant differences of food intake, body weight, heart rate, SBP or DBP, Total-C, HDL-C, triglycerides, insulin, Total-GIP, FBG and HbA1c among 4 groups.

### 3.2. Effects of [D-Ala^2^]GIP(1–42) on Foam Cell Formation of, and CD36 Expression in, Macrophages Isolated from Gipr^−/−^ Mice and Gipr^+/+^ Mice

**Figure 1:**
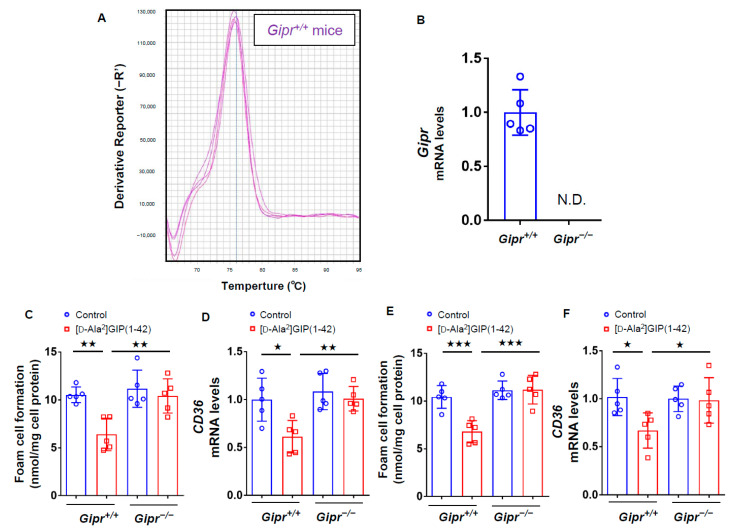
Effects of [D-Ala^2^]GIP(1–42) on foam cell formation and *CD36* gene expression in *Gipr^−/−^* mice and *Gipr^+/+^* mice. Peritoneal macrophages were isolated from *Gipr^−/−^* mice and *Gipr^+/+^* mice infused subcutaneously with or without [D-Ala^2^]GIP(1–42) at 25 nmol/kg/day for 4 weeks. The cells were incubated with 10 μg/mL ox-LDL containing at 0.1 mmol/L [^3^H]oleate in RPMI 1640 medium supplemented with 10% fetal bovine serum (FCS) containing 100 U/mL penicillin and 100 μg/mL streptomycin at 37 °C in 5% CO_2_ for 18 h. (**A**) Melt curve of gene expression of *Gipr* in peritoneal macrophages extracted from *Gipr^+/+^* mice. (**B**) Gene expression levels of *Gipr* in peritoneal macrophages from *Gipr^−/−^* mice and *Gipr^+/+^* mice. (**C**,**D**) Foam cell formation evaluated by the radioactivity of cholesterol [^3^H]oleate (**C**) and *CD36* gene expression levels (**D**) in macrophages isolated from *Gipr^−/−^* and *Gipr^+/+^* mice infused subcutaneously with or without [D-Ala^2^]GIP(1–42). (**E**,**F**) Macrophages were first extracted from *Gipr^−/−^* and *Gipr^+/+^* mice, and then exposed to [D-Ala^2^]GIP(1–42) at 1 nmol/L. Foam cell formation (**E**) and CD36 gene expression (**F**) were evaluated. Total RNAs were transcribed and amplified by real-time PCR. Values were normalized by the intensity of glyceraldehyde 3-phosphate dehydrogenase *(GAPDH)* mRNA-derived signals and then compared to the control intensities. Number = 5 for each group. Data are presented as mean ± standard deviation. The significant value was shown as ^★★★^ *p* < 0.005, ^★★^ *p* < 0.01 and ^★^ *p* < 0.05.

We first investigated the effects of GIP on foam cell formation of, and *CD36* gene expression in, macrophages by using *Gipr^−/−^* mice and *Gipr^+/+^* mice subcutaneously infused with or without [D-Ala^2^]GIP(1–42) at 25 nmol/kg/day for 4 weeks. As shown in Figure 1A,B, *Gipr* gene was actually expressed in peritoneal macrophages extracted from *Gipr^+/+^* mice, while it was not detected in *Gipr^−/−^* mice (Figure 1A,B). Foam cell formation measured by the radioactivity of cholesterol [^3^H]oleate and *CD36* expression in macrophages isolated from *Gipr^+/+^* mice infused subcutaneously with [D-Ala^2^]GIP(1–42) were significantly suppressed compared with vehicle-infused mice, while these beneficial effects were not observed in macrophages isolated from *Gipr^−/−^* mice infused with [D-Ala^2^]GIP(1–42) (Figure 1C,D). When macrophages were first extracted from *Gipr^−/−^* mice and *Gipr^+/+^* mice, and then exposed to [D-Ala^2^]GIP(1–42), [D-Ala^2^]GIP(1–42) at 1 nmol/L significantly inhibited the foam cell formation of, and *CD36* gene expression in, macrophages derived from *Gipr^+/+^* mice, but not from *Gipr^−/−^* (Figure 1E,F).

### 3.3. Effects of [D-Ala^2^]GIP(1–42) and (R)-DRF053 on U937 Macrophages

**Figure 2:**
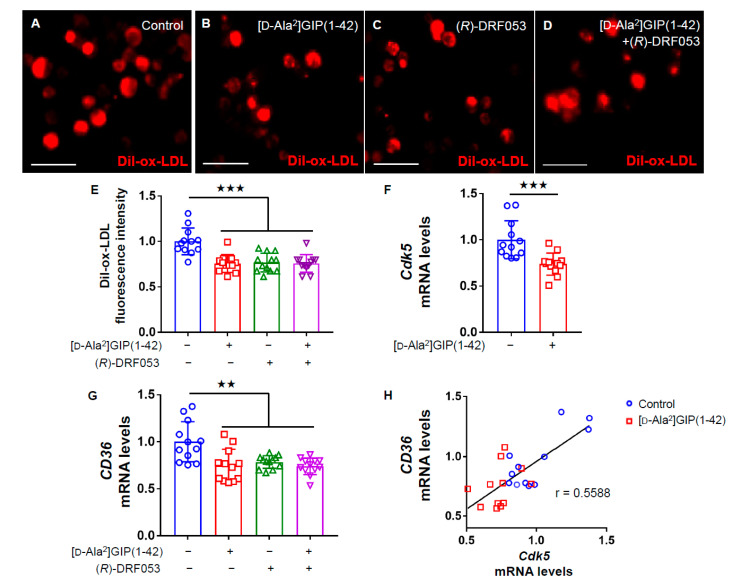
Effects of [D-Ala^2^]GIP(1–42) and (*R*)-DRF053 dihydrochloride on Dil-ox-LDL uptake, *Cdk5* and *CD36* gene expression in U937 cells. U937 were incubated with 10 μg/mL Dil-ox-LDL with or without 1 nmol/L [D-Ala^2^]GIP(1–42) or 0.215 µmol/L, a selective inhibitor of Cdk5 (*R*)-DRF053 dihydrochloride in RPMI 1640 medium supplemented with 10% FCS containing 100 U/mL penicillin and 100 μg/mL streptomycin at 37 °C for 18 h. (**A**–**D**) Representative immunofluorescent staining images in human U937 macrophages. Dil-ox-LDL-positive cells were stained in red. Scale bars, 50 µm. (**E**) Quantification of fluorescence intensity in red. Dil-ox-LDL uptake was normalized by the control values. (**F**–**H**) Gene expression levels of *Cdk5* (**F**), and *CD36* (**G**), and their correlation (**H**). Total RNAs were transcribed and amplified by real-time PCR. Data were normalized by the value of *GAPDH* mRNA-derived signals and then related to the control intensities. Number = 12 for each group. Error bars are standard deviation. ^★★★^ *p* < 0.005, ^★★^ *p* < 0.01 vs. control.

We then examined the effects of [D-Ala^2^]GIP(1–42) on macrophage foam cell formation evaluated by uptake of Dil-ox-LDL into U937 cells. Immunofluorescent staining images revealed that [D-Ala^2^]GIP(1–42) significantly decreased the intensity of Dil-ox-LDL-positive cells (Figure 2A–E). Furthermore, as shown in Figure 2F,G, *Cdk5* and *CD36* gene expression levels were significantly suppressed by [D-Ala^2^]GIP(1–42) in U937 cells, whereas a selective inhibitor of Cdk5, (*R*)-DRF053 dihydrochloride mimicked the effects of [D-Ala^2^]GIP(1–42) on U937 macrophages. In addition, no additive combination effects of Cdk5 inhibitor and [D-Ala^2^]GIP(1–42) on *CD36* expression and Dil-ox-LDL uptake were observed (Figure 2E,G). Furthermore, there was a significant correlation between *Cdk5* and *CD36* gene expression levels (Figure 2H).

## 4. Discussion

We have previously reported that chronic infusion of active GIP(1–42) at 25 nmol/kg/day, the same concentration used in the present experiments, significantly suppresses the foam cell formation of macrophages and subsequent progression of atherosclerosis in *Apoe^−/−^* mice [14,15]. However, the underlying molecular mechanisms for this remain largely unclear. To address the issue, we first examined the effects of [D-Ala^2^]GIP(1–42), a dipeptidyl peptidase-4-resistant GIP receptor agonist on foam cell formation of, and *CD36* gene expression in, macrophages isolated from *Gipr^−/−^* and *Gipr^+/+^* mice. We found here that subcutaneously long-term infusion of [D-Ala^2^]GIP(1–42) to mice significantly inhibited the foam cell formation evaluated by the radioactivity of cholesterol [^3^H]oleate and *CD36* gene expression in macrophages isolated from *Gipr^+/+^* mice compared with vehicle-treated mice, while these beneficial effects of [D-Ala^2^]GIP(1–42) were not observed in macrophages isolated from *Gipr^−/−^* mice. These observations suggest that the inhibitory effects of [D-Ala^2^]GIP(1–42) on foam cell formation of, and *CD36* gene expression in, mouse macrophages could be mediated through the interaction with GIP receptor. CD36 is one of the main scavenger receptors, which could regulate foam cell formation of macrophages, the early characteristic features of atherosclerosis [3,5]. We have recently reported that (1) neutralizing anti-CD36 antibody inhibits ox-LDL uptake into AGE-exposed U937 macrophages [25] and (2) [D-Ala^2^]GIP(1–42) attenuates cholesterol accumulation of macrophages in *Apoe^−/−^* mice in association with the reduction of *CD36* expression [14,15]. These observations suggest that the suppressive effect of [D-Ala^2^]GIP(1–42) on foam cell formation could be mediated by reduction of *CD36* gene expression in macrophages.

Cdk5 is constitutively expressed in macrophages, which could contribute to inflammatory reactions in these cell types [19]. In this study, gene expression levels of *Cdk5* and *CD36* were significantly suppressed by [D-Ala^2^]GIP(1–42) in U937 macrophages, and these gene expression levels were correlated with each other. Since (1) a selective inhibitor of Cdk5 mimicked the effects of [D-Ala^2^]GIP(1–42) on U937 macrophages and (2) no additive combination effects of Cdk5 inhibitor and [D-Ala^2^]GIP(1–42) were observed, reduction of foam cell formation and *CD36* gene expression, [D-Ala^2^]GIP(1–42) may inhibit foam cell formation of macrophages through the suppression of Cdk5-CD36 pathway via the interaction with GIP receptor. Recently, we have found that (*R*)-DRF053, a selective inhibitor of Cdk5 significantly inhibits foam cell formation of AGE-exposed macrophages by reducing *CD36* gene expression [25]. These findings suggest that reduction of *Cdk5* gene expression by [D-Ala^2^]GIP(1–42) may contribute to its suppressive effects on macrophage foam cell formation.

**Figure 3:**
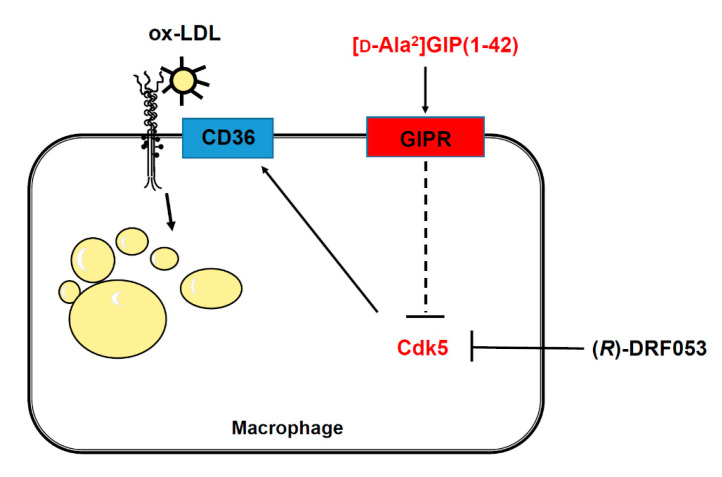
Possible mechanisms how [D-Ala^2^]GIP(1–42) could inhibit foam cell formation of macrophages. [D-Ala^2^]GIP may suppress macrophage foam cell formation through the transcriptional inhibition of CD36 via Cdk5 pathway. GIP, glucose-dependent insulinotropic polypeptide; GIPR, receptor of glucose-dependent insulinotropic polypeptide; Cdk5, cyclin-dependent kinase 5; ox-LDL, oxidized low-density lipoprotein.

Interaction of GIP with the GIP receptor induces activation of adenosine monophosphate-activated protein kinase (AMPK) through the phospholipase C and calcium/calmodulin-dependent protein kinase pathway [11,41,42,43]. Recently, crocin, a carotenoid compound, has been found to activate AMPK and subsequently improve metabolic dysfunction in diabetic mice via the suppression of *Cdk5* expression [44,45]. These observations suggest that GIP and its receptor interaction may inhibit macrophage foam cell formation by suppressing the Cdk5-CD36 pathway via the activation of AMPK (Figure 3).

Our study has some potential limitations. First, [D-Ala^2^]GIP(1–42) is a human type of GIP agonist. Amino acid sequences of human GIP(1–42) is almost identical to that of mouse [46]. This could support the compatibility of [D-Ala^2^]GIP(1–42) in mouse experiments. Second, (*R*)-DRF053 is not a specific selective inhibitor of Cdk5, but it inhibits Cdk1 and other Cdk members among other kinases. However, we cannot obtain an additional structure unrelated inhibitor of Cdk5. It would be interesting to examine the effects of [D-Ala^2^]GIP(1–42) on *CD36* gene expression and form cell formation of macrophages in *Cdk5*-knockout mice. Third, *Cdk5* gene expression in the mice was not examined in the present experiments because of the lack of samples. Therefore, although an inhibitor of Cdk5, (*R*)-DRF053 inhibited *CD36* mRNA levels in U937 cells and that there was a positive correlation between *Cdk5* and *CD36* mRNA levels (Figure 2F–H), the conclusion that inhibition of the Cdk5-CD36 pathway via the GIP receptor suppresses macrophage foam cell formation cannot be definitely proved without confirmation in mouse experiments. Fourth, protein expression levels of CD36 were not evaluated, however we found that *CD36* gene expression and foam cell formation in mouse and human macrophages were correlated with each other. Therefore, CD36 protein expression could be functionally correlated with foam cell formation of macrophages [5], and *CD36* gene expression leads to reflect surface cellular expression of the protein. Additionally, some reports showed that Cdk5 activity is highly correlated with level of *Cdk5* gene expression [17,47]. Finally, it would be interesting to examine the effects of AMPK inhibitor on foam cell formation of [D-Ala^2^]GIP(1–42)-exposed U937 cells.

## 5. Conclusions

We found here that [D-Ala^2^]GIP(1–42) could inhibit foam cell formation of macrophages through Cdk5-CD36 pathway via GIP receptor. Inhibition of Cdk5-CD36 pathway by GIP in macrophage may be a novel therapeutic target for atherosclerotic cardiovascular disease.

## Figures and Tables

**Table 1:** Laboratory characteristics of *Gipr^−/−^* mice and *Gipr^+/+^* mice at 13 weeks old.

	*Gipr^+/+^* Mice	*Gipr^+/+^* Mice +[D-Ala^2^]GIP(1–42)	*Gipr^−/−^* Mice	*Gipr^−/−^* Mice +[D-Ala^2^]GIP(1–42)
Number	5	5	5	5
Final body weight (g)	24 ± 0.8	24.1 ± 1.7	22.3 ± 1.4	22.5 ± 0.7
Food Intake (g/day)	4.0 ± 0.7	4.1 ± 1.0	4.3 ± 0.5	4.3 ± 0.6
SBP (mmHg)	102 ± 11	100 ± 8	102 ± 8	103 ± 11
DBP (mmHg)	63 ± 9	58 ± 4	65 ± 9	62 ± 10
Heart rate (bpm)	538 ± 50	561 ± 55	591 ± 48	594 ± 51
Total-C (mg/dL)	104 ± 10	106 ± 4	112 ± 10	117 ± 23
HDL-C (mg/dL)	36 ± 16	41 ± 7	53 ± 12	49 ± 12
Triglycerides (mg/dL)	99 ± 8	103 ± 7	52 ± 43	58 ± 44
Insulin (ng/mL)	0.4 ± 0.25	0.41 ± 0.13	0.43 ± 0.2	0.46 ± 0.12
Total-GIP (pmol/L)	30 ± 8	46 ± 18	34 ± 5	43 ± 7
FBG (mg/dL)	99 ± 8	98 ± 9	100 ± 21	101 ± 9
HbA1c (%)	4.7 ± 0.2	4.8 ± 0.2	4.9 ± 0.1	4.9 ± 0.2

GIP, glucose-dependent insulinotropic polypeptide; SBP, systolic blood pressure; DBP, diastolic blood pressure; Total-C, total-cholesterol; HDL-C, high-density lipoprotein cholesterol; FBG, fasting blood glucose; HbA1c, glycated hemoglobin; Results are presented as mean values ± standard deviation. The significant value was shown as *p* < 0.05 vs. each group.

## Data Availability

All data used in this study are available from the corresponding author on reasonable request.
